# Platelet dysfunction contributes to bleeding complications in patients with probable leptospirosis

**DOI:** 10.1371/journal.pntd.0005915

**Published:** 2017-09-21

**Authors:** Rahajeng N. Tunjungputri, Muhammad Hussein Gasem, Willemijn van der Does, Pandu H. Sasongko, Bambang Isbandrio, Rolf T. Urbanus, Philip G. de Groot, Andre van der Ven, Quirijn de Mast

**Affiliations:** 1 Department of Internal Medicine, Radboud university medical center, Nijmegen, The Netherlands; 2 Center for Tropical and Infectious Disease (CENTRID), Faculty of Medicine Diponegoro University, Dr Kariadi Hospital, Semarang, Indonesia; 3 National Reference Laboratory for Leptospira, Dr. Kariadi Hospital, Semarang, Indonesia; 4 Department of Clinical Chemistry and Haematology, University Medical Center, Utrecht, The Netherlands; University of California Davis, UNITED STATES

## Abstract

**Background:**

Severe leptospirosis is frequently complicated by a hemorrhagic diathesis, of which the pathogenesis is still largely unknown. Thrombocytopenia is common, but often not to the degree that spontaneous bleeding is expected. We hypothesized that the hemorrhagic complications are not only related to thrombocytopenia, but also to platelet dysfunction, and that increased binding of von Willebrand factor (VWF) to platelets is involved in both platelet dysfunction and increased platelet clearance.

**Methodology/Principal findings:**

A prospective study was carried out in Semarang, Indonesia, enrolling 33 hospitalized patients with probable leptospirosis, of whom 15 developed clinical bleeding, and 25 healthy controls. Platelet activation and reactivity were determined using flow cytometry by measuring the expression of P-selectin and activation of the α_IIb_β_3_ integrin by the binding of fibrinogen in unstimulated samples and after *ex vivo* stimulation by the platelet agonists adenosine-diphosphate (ADP) and thrombin-receptor activating peptide (TRAP). Platelet-VWF binding, before and after VWF stimulation by ristocetin, as well as plasma levels of VWF, active VWF, the VWF-inactivating enzyme ADAMTS13, thrombin-antithrombin complexes (TAT) and P-selectin were also measured. Bleeding complications were graded using the WHO bleeding scale. Our study revealed that platelet activation, with a secondary platelet dysfunction, is a feature of patients with probable leptospirosis, especially in those with bleeding manifestations. There was a significant inverse correlation of bleeding score with TRAP-stimulated P-selectin and platelet-fibrinogen binding (R = -0.72, P = 0.003 and R = -0.66, P = 0.01, respectively) but not with platelet count. Patients with bleeding also had a significantly higher platelet-VWF binding. Platelet counts were inversely correlated with platelet-VWF binding (R = -0.74; *P* = 0.0009. There were no correlations between platelet-VWF binding and the degree of platelet dysfunction, suggesting that increased platelet-VWF binding does not directly interfere with the platelet α_IIb_β_3_ signaling pathway in patients with probable leptospirosis.

**Conclusion/Significance:**

Platelet dysfunction is common in probable leptospirosis patients with manifest bleeding. Increased VWF-platelet binding may contribute to the activation and clearance of platelets.

## Author summary

Bleeding is a frequent complication of leptospirosis, a disease caused by the pathogenic spirochaete *Leptospira*. Although thrombocytopenia is common, studies have shown that it does not fully explain the bleeding events seen in these patients. We hypothesized that platelet dysfunction plays a role in the development of bleeding complications. The present study involved 33 hospitalized patients with probable leptospirosis and 25 healthy controls. We report that circulating platelets from patients with severe, probable leptospirosis were activated, but less reactive to *ex vivo* activation. The degree of this platelet dysfunction was associated with bleeding, in contrast to the degree of thrombocytopenia. Platelets of leptospirosis patients also demonstrated increased binding with von Willebrand factor (VWF), and a strong negative correlation with platelet count suggesting that this binding is important for platelet clearance.

## Introduction

Leptospirosis is a zoonotic disease of global importance caused by the pathogenic spirochaetes of the genus *Leptospira* [1, 2]. A modeling exercise by the World Health Organization's (WHO's) Leptospirosis Burden Epidemiology Group, estimated that 873,000 cases and 48,600 deaths occur worldwide each year [3]. The clinical manifestations of leptospirosis range from a mild, self-limited febrile illness to a fulminant life-threatening illness with multi-organ failure [1, 2]. Bleeding complications are common in severe leptospirosis, being reported in up to 60% of all hospitalized patients. Although the majority of bleeding events are mild, some patients may develop severe gastrointestinal or pulmonary hemorrhage, the latter having an alarmingly high mortality of >50% [4, 5].

The pathophysiological mechanisms responsible for bleeding remain incompletely understood. Thrombocytopenia is frequently observed and is associated with poor outcome [6], but its severity is not to the extent that spontaneous bleeding is expected [7]. Platelet dysfunction might also contribute to bleeding. Measuring platelet function in thrombocytopenic conditions is technically demanding as commonly used techniques such as light transmission aggregometry are not useful [8]. To the best of our knowledge, no studies on platelet function in leptospirosis have been performed.

Platelets are involved in primary hemostasis, wherein they aggregate and form a plug to seal off vascular leakage. Platelet adhesion is initiated by the binding of GPIbα and GPVI receptors on platelets to VWF on endothelial surface and collagen, respectively, of which both are exposed from the subendothelium after vascular injury. Platelets are then activated, and adhesion and aggregation is strengthened via the platelet αIIbβ3 receptor binding with fibrinogen and von Willebrand factor (VWF).

The binding of VWF to the glycoprotein (GP)-1b receptor on platelets is a well-known platelet clearance mechanism [9]. VWF is a multimeric glycoprotein that predominantly originates from the endothelium. Its main function is the recruitment of platelets to sites of vascular injury. Under normal conditions, most of the VWF circulates in non-platelet binding form. VWF may however undergo a conformational change to a more active, platelet-binding form facilitating platelet aggregation [10]. An increase in this so-called ‘active’ VWF has been described in a number of infectious and non-infectious diseases [11–13]. A deficiency in ADAMTS13 (a disintegrin and metalloproteinase thrombospondin type 1 motif, member 13), an enzyme that cleaves large VWF multimers into their inactive conformation, may also lead to excessive intravascular platelet agglutination [14].

Von Willebrand disease type 2B is characterized by excessive VWF binding to platelets. Recently, it was reported that this excessive binding not only leads to thrombocytopenia, but also to thrombocytopathy via inhibition of the platelet α_IIb_β_3_, an integrin that primarily mediates platelet aggregation through binding of fibrinogen [15]. In addition, excessive platelet activation may also lead to exhausted and hence dysfunctional platelets [16]. We previously found in patients with dengue hemorrhagic fever that circulating platelets were activated, but less reactive to *ex vivo* stimulation with platelet agonists [17]. We hypothesized that similar platelet changes also occur in other hemorrhagic infectious diseases, including in severe leptospirosis. Specifically, we hypothesized that circulating platelets in severe leptospirosis are activated and have bound more VWF and that both these processes contribute to thrombocytopenia and platelet dysfunction, especially in those with bleeding complications. We therefore characterized VWF binding to platelets, platelet activation and platelet reactivity using flow cytometry in adult Indonesian patients with probable leptospirosis and determined whether these parameters were related to bleeding complications. In addition, we determined the plasma levels of VWF, active VWF, P-selectin, the plasma coagulation factor thrombin-antithrombin complexes (TAT) and ADAMTS13 activity.

## Methods

### Ethics statement

The study was approved by the Ethics Committee of the Faculty of Medicine Diponegoro University and Dr Kariadi Hospital, Semarang, Indonesia. Upon participation, all participants signed an informed consent form.

### Study population

We carried out a prospective study at the Department of Internal Medicine of Dr. Kariadi Hospital, Semarang, Indonesia, between December 2013-March 2014. We enrolled adult patients admitted with a high clinical suspicion of leptospirosis using the case definition of the World Health Organization South East Asia Regional Office supported by a positive result of Leptospira IgM lateral flow (Pakar Biomedika, Indonesia), classifying them as probable leptospirosis [18]. The Leptospira IgM lateral flow test had a reported sensitivity and specificity of 85.6% and 96.2%, respectively [19], while another study by Goris *et*. *al*. reported a specificity of 95% [20]. Clinical diagnosis of leptospirosis was confirmed in a single acute sample by microscopic agglutination test (MAT) with a panel consisting of 31 serovars (28 pathogenic serovars: Australis, Bratislava, Autumnalis, Rachmati, Ballum, Castellonis, Bataviae, Benjamini, Whitcombi, Cynopteri, Grippotyphosa, Hebdomadis, Copenhageni, Hardjo, Icterohaemorrhagiae, Lai, Naam, Coxi, Javanica, Panama, Pomona, Proechimys, Pyrogenes, Sarmin, Saxkoebing, Sejroe, Shermani, Tarassovi; three non-pathogenic serovars: Andamana, Patoc and Semaranga). A titre of ≥1/320 on a single sample was considered positive. Additionally, dengue IgM/IgG serology was tested in all patients as part of the protocol in patients with clinical suspicion of leptospirosis in the hospital where this study was performed, and patients with a positive dengue IgM were not enrolled. Healthy controls were all tested negative for rapid Leptospira IgM lateral flow. All diagnostic tests were performed at the National Reference Laboratory for Leptospira, Dr. Kariadi Hospital, Semarang, Indonesia. Patients and healthy controls were included after written informed consent was obtained. Blood samples were obtained on inclusion day (day 1) and upon follow up (day 4). The study is approved by the ethical committee of the Faculty of Medicine Diponegoro University-Dr. Kariadi Hospital, Semarang, Indonesia.

### Platelet activation and reactivity and platelet-von Willebrand factor binding

Whole blood was collected from patients by using venipuncture from the antecubital vein into citrate-anticoagulated tubes (3.2%; BD Vacutainer, Becton Dickinson). All samples were processed within one hour after blood collection. Platelet activation and reactivity were determined by flow cytometry using a method previously described [21, 22]. In short, the platelet membrane expression of P-selectin (CD62P) and platelet-fibrinogen binding, which correspond with platelet degranulation and aggregation, were determined in unstimulated whole blood and in whole blood stimulated 20 min with the platelet agonists adenosine-diphosphate (ADP; 7.8 μM and 31.2 μM) or thrombin receptor-activating peptide (TRAP; 39 μM and 625 μM) diluted in HEPES-buffered saline. Saturating concentrations of the following monoclonal antibodies were used: PE-labeled anti-CD62P (Bio-Legend, San Diego, USA), FITC-labeled anti-fibrinogen (DAKO Ltd., High Wycombe, UK), and PC7-labeled anti-CD61 (platelet identification marker; Beckman Coulter, France). Platelets were gated based on their forward- and sideward-scatter (FSC/SSC) properties and positivity for CD61, which was defined as a median fluorescence intensity (MFI) exceeding the MFI of the matched isotype control.

The binding of VWF to platelets was determined by adding whole blood sample to a mixture of HEPES-buffered saline and saturating concentrations of FITC-labeled anti-von Willebrand factor (Abcam, Cambridge, UK) and PC7-labeled anti-CD61, with or without the addition of ristocetin (0,84 mg/ml and 1,5 mg/ml). After incubation for 20 minutes at room temperature, a fixative solution (0.2% paraformaldehyde) was added and samples were analyzed with a BD FACS Canto II flow cytometer (Becton Dickinson, USA). Next, the MFI of CD62P, fibrinogen and VWF on CD61-positive events were determined.

### Plasma markers

Platelet-poor plasma was obtained from citrate-anticoagulated whole blood by centrifugation (1500 g without brake, 15 min, 20ehyde) was added and thrombin-antithrombin (TAT) complexes were subsequently measured with enzyme-linked immunosorbent assay (ELISA) as previously described [24]. Sheep anti-human thrombin (SAHT-AP, SAHT-HRP) antibodies were purchased from Kordia/Affinity Biologicals, USA. Plasma VWF concentrations were determined with ELISA as described previously [23]. Active VWF was quantified by ELISA using a nanobody (AU/VWFa-11) that recognizes the GPIb binding configuration of VWF, as described previously [24]. We express the relative amount of VWF that circulates in its active, platelet binding conformation by using the term VWF activation factor. VWF activation factor of normal pooled plasma was referred to as 1. ADAMTS13 activity was quantified using the fluorescence resonance energy transfer (FRETS) assay (Peptides International, Lexington, USA), whereby the ADAMTS13 activity of normal pool plasma was set at 100%, and the values obtained in study participant samples were expressed as percentage of normal pool plasma [25].

### Statistical analysis

Differences in subject characteristics were compared with analysis of variance (ANOVA) with posttests for comparing more than two groups, and with Mann-Whitney U-test or chi-square test for comparing the two patient groups. Data are presented as median with interquartile range (IQR) unless stated otherwise. Relationships between parameters were assessed with the Spearman correlation coefficient. All analyses were performed with SPSS version 20 (SPSS, Inc., Chicago, Illinois, USA). *P* values less than 0.05 were considered statistically significant.

## Results

### Patient characteristics

Thirty-three consecutive hospitalized patients with probable leptospirosis were enrolled together with 25 healthy controls. Twelve (36%) of these patients had a positive result of the MAT (titer >1/320). All patients were tested negative for dengue IgM, while 29 had a positive dengue IgG (12 in the bleeders group and 17 in the non-bleeders group). Characteristics of participants are presented in Table 1. Fifteen patients (46%) presented with or developed clinical relevant bleeding manifestations during hospitalization. The most common were gastrointestinal (n = 10) and genitourinary (n = 8) bleeding events. The severity of bleeding events in patients was graded using the WHO bleeding scale [26–28]. Ten out of 15 patients (67%) who experienced clinical bleeding developed Grade 2 bleeding (i.e. epistaxis with a total duration of all episodes in previous 24 hours of >30 minutes, grossly visible blood in urine, stool or emesis), while 4 (27%) developed that of Grade 1 (i.e. epistaxis with a total duration in previous 24 hours of <30 minutes, microscopic hematuria and petechiae). There was one patient (7%) with Grade 3 bleeding whose hematemesis and melena caused hemodynamic instability. One Grade 1 (7%) and three (20%) Grade 2 patients died during hospitalization.

**Table 1:** 

	All patients	Bleeders	Non-bleeders	Reference values	Controls	*P* value
Numbers (%)	33	15 (46)	18 (54)		25	
Male sex, n (%)	22 (67)	7 (46.7)	15 (83)		17 (68)	0.03
Age (years)	47 (36–53)	52 (38–59)	48 (40–52)		36 (32–50)	0.24
Bleeding events, n (%)						
Oral, nasal, skin						
Epistaxis		2 (6)				
Petechiae		2 (6)				
Gastrointestinal						
Hematochezia		1 (3)				
Hematemesis		5 (15)				
Melena		4 (12)				
Genitourinary						
Microscopic hematuria		3 (9)				
Gross hematuria		5 (15)				
Days post-onset illness, n	8 (7–11)	7 (6–10)	7 (7–12)			0.12
MAT positive (%)	12 (36)	9 (60)	3 (17)			0.02
Hb (gr/dl)	13.3 (10.8–15)	10.9 (9.4–13.2)	14.8 (13.9–16.5)	12–15		0.004
Platelet count (x10^9^/l)	114 (60–172)	105 (56–152)	123 (106–183)	150–300		0.003
Thrombocytopenia <150x10^9^/l (%)	17 (52)	10 (67)	7 (39)			0.11
Thrombocytopenia <100x10^9^/l (%)	9 (27)	7 (47)	2 (11)			0.03
Leukocyte count (x10^9^/l)	14.5 (9.4–17.4)					
Bilirubin (mg/dl)	1.6 (0.8–6.4)	2.0 (1.5–5.2)	1.3 (0.8–5.3)	0–1.0		0.28
AST (U/l)	89 (44–147)	84 (58–131)	95 (46–138)	15–35		0.72
ALT (U/l)	93 (63–160)	87 (62–131)	99 (79–193)	15–60		0.68
Urea (mg/dl)	47 (31–196)	64 (46–221)	36 (24–49)	15–39		0.06
Creatinine (mg/dl)	1.7 (1.1–4.6)	2.0 (1.4–5.1)	1.4 (1–2)	0.6–1.3		0.12

Numbers represent median (IQR) or number (%) of patients. *P* values represent difference between bleeders and non-bleeders (Mann-Whitney U-test or chi-square test when appropriate). A *P* value <0.05 was considered statistically significant.

Median (IQR) platelet count was significantly lower in the group of bleeders compared with the non-bleeders (105, 56-152x10^9^/l vs. 123, 106–183 x10^9^/l; *P* = 0.003). Five patients had a platelet count lower than 50 x10^9^/l (platelet counts of four bleeders: 12 x10^9^/l, 17 x10^9^/l, 30 x10^9^/l, 30 x10^9^/l; platelet count of one non-bleeder: 49 x10^9^/l). Additionally, the hemoglobin levels were lower in the bleeders group than the non-bleeders group (10.9, 9.4–13.2 gr/dl vs. 14.8, 13.9–16.5 gr/dl; *P* = 0.004), with six patients having hemoglobin levels below 10gr/dl (hemoglobin levels of five bleeders: 8.2 gr/dl, 8.9 gr/dl, 9.1 gr/dl, 9.4 gr/dl, 9.8 gr/dl; hemoglobin level of one non-bleeder: 9.5 gr/dl). Leukopenia is a common presentation of dengue. However, none of the patients in our patient cohort had leukopenia at presentation, with a median (interquartile range) of leukocyte of 14.5 x109/l (9.4 x109/l -17.4 x109/l).

### Bleeding is associated with higher platelet activation and platelet dysfunction

**Fig 1:**
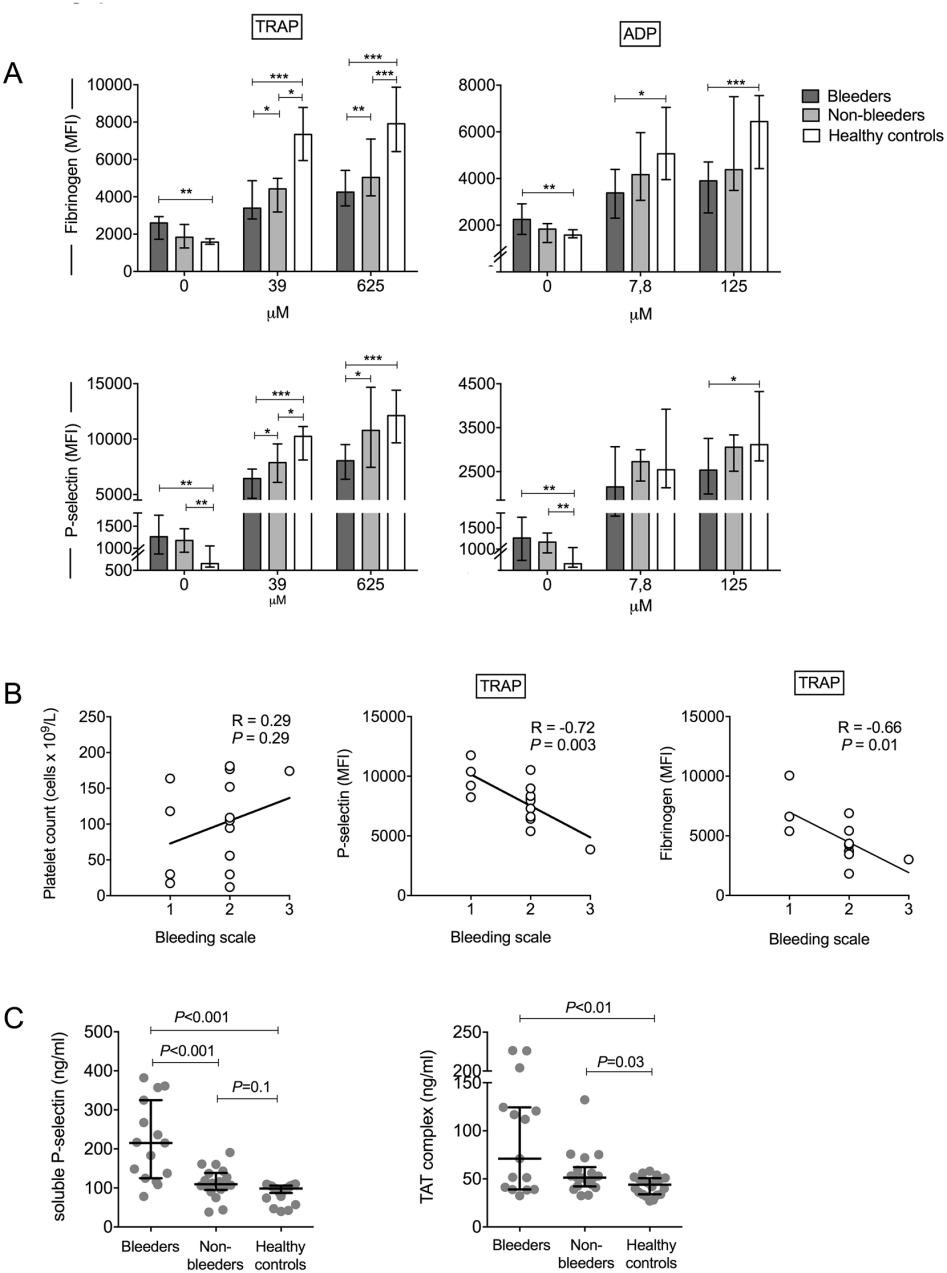
Platelet activation and reactivity, plasma P-selectin and TAT complexes and the relationship between bleeding and platelet number and reactivity. (A) Platelet-fibrinogen binding and the platelet membrane expression of P-selectin (depicted as median fluorescence intensity (MFI) in arbitrary units) in unstimulated samples and after stimulation with two concentrations of the platelet agonists thrombin receptor activating peptide (TRAP) and adenosine diphosphate (ADP) in leptospirosis patients with bleeding (bleeders, n = 15) and without bleeding (non-bleeders, n = 18) and in healthy controls (n = 25). Data depicted are medians with interquartile ranges (IQR). * *P*<0.05, **P<0.005, ***P<0.001. (B) Correlations between the severity of bleeding, as measured using the WHO bleeding scale, and platelet count, as well as TRAP-induced (625 μM) platelet P-selectin expression and platelet-fibrinogen binding. Shown are the Spearman correlation coefficients. (C) Plasma concentration of soluble P-selectin and thrombin-antithrombin (TAT) complex. The lines with error bars indicate medians with IQR.

Fig 1A shows the fibrinogen binding to the activated α_IIb_β_3_ receptor and expression of P-selectin on platelets. Patients with bleeding had significantly higher platelet-fibrinogen binding (MFI 2601, 1726–2938 vs. 1577, 1460–1752; *P* = 0.001) and P-selectin (MFI 1095, 798–1685 vs. 654, 575–1056; *P* = 0.002) in unstimulated blood samples than controls, suggesting increased platelet activation. The non-bleeding patients also had significantly higher membrane P-selectin expression compared to controls (MFI 1179, 910–1444; *P* = 0.003). Platelet reactivity was assessed by *ex vivo* stimulation of whole blood with two concentrations of the platelet agonists ADP or TRAP. In contrast to the findings in unstimulated samples, P-selectin expression and fibrinogen binding in response to TRAP or ADP were lower in the bleeders compared with non-bleeders and controls, suggestive of platelet dysfunction. A reduction in platelet reactivity, albeit to a lesser extent, was also found in the non-bleeders in response to TRAP.

Interestingly, whereas bleeding score was not associated with platelet count (Fig 1B), an inverse relation was present for bleeding score with P-selectin expression and fibrinogen binding to *ex vivo* stimulation with TRAP (R = -0.72; *P* = 0.003 for P-selectin and R = -0.66; *P* = 0.01 for fibrinogen; Fig 1B) and ADP (R = -0.41; *P* = 0.01 and R = -0.63; *P* = 0.03, respectively; shown in S1 Fig).

The flow cytometric findings of platelet activation in bleeders were supported by measurement of plasma concentrations of soluble P-selectin, which was significantly higher in bleeders compared to both the non-bleeders and healthy controls. Plasma P-selectin in the non-bleeders was comparable with that of the controls. Overall, leptospirosis patients also had significantly higher plasma TAT concentrations, suggesting activation of the plasma coagulation pathway, with the highest concentrations found in the bleeders (Fig 1C).

**Fig 2:**
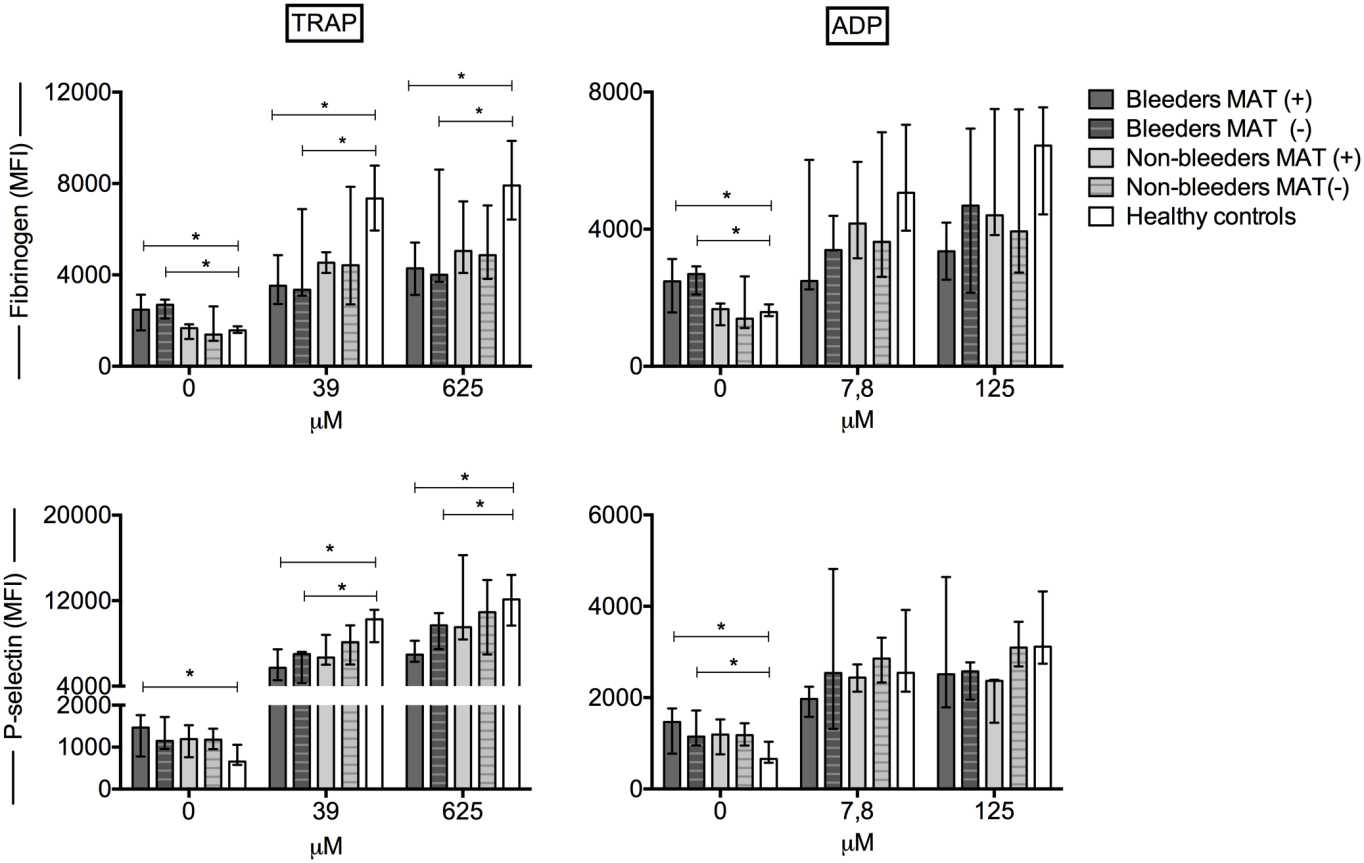
Platelet activation and reactivity in patient groups based on results of the microscopic agglutination test (MAT). Platelet-fibrinogen binding and the platelet membrane expression of P-selectin (depicted as median fluorescence intensity (MFI) in arbitrary units) in unstimulated samples and after stimulation with two concentrations of the platelet agonists thrombin receptor activating peptide (TRAP) and adenosine diphosphate (ADP) in MAT-positive leptospirosis patients with bleeding (bleeders, n = 15; MAT(+), n = 9; MAT(-), n = 6) and without bleeding (non-bleeders, n = 18; MAT(+), n = 3; MAT(-), n = 15) and in healthy controls (n = 25). Data depicted are medians with IQR. * *P*<0.05.

Comparing MAT-positive with MAT-negative patients, no differences were found in platelet activation status as well as platelet reactivity in the bleeders and non-bleeders (Fig 2). There was a trend for lower platelet count in the MAT-positive group, with a median (IQR) of 135 x10^9^/l (50 x10^9^/l -180 x10^9^/l), compared to the MAT-negative group (134 x10^9^/l, 105 x10^9^/l -244 x10^9^/l; *P* = 0.09). Additionally, there were no statistically significant differences in the bilirubin, AST, ALT, ureum and creatinine levels between the MAT-positive and MAT-negative patients. In further analyses, MAT-positive and MAT-negative cases were analyzed as a single group of bleeders or non-bleeders.

Follow-up data at day four were available for 11 bleeders and 10 non-bleeders. Bleeders, but not the non-bleeders, remained having a significantly reduced platelet reactivity in the follow-up on day 4 compared to healthy controls (S2A Fig). Plasma P-selectin also remained significantly increased at day 4 in bleeders (S2B Fig).

### Platelet von-Willebrand factor binding was highest in bleeders

**Fig 3:**
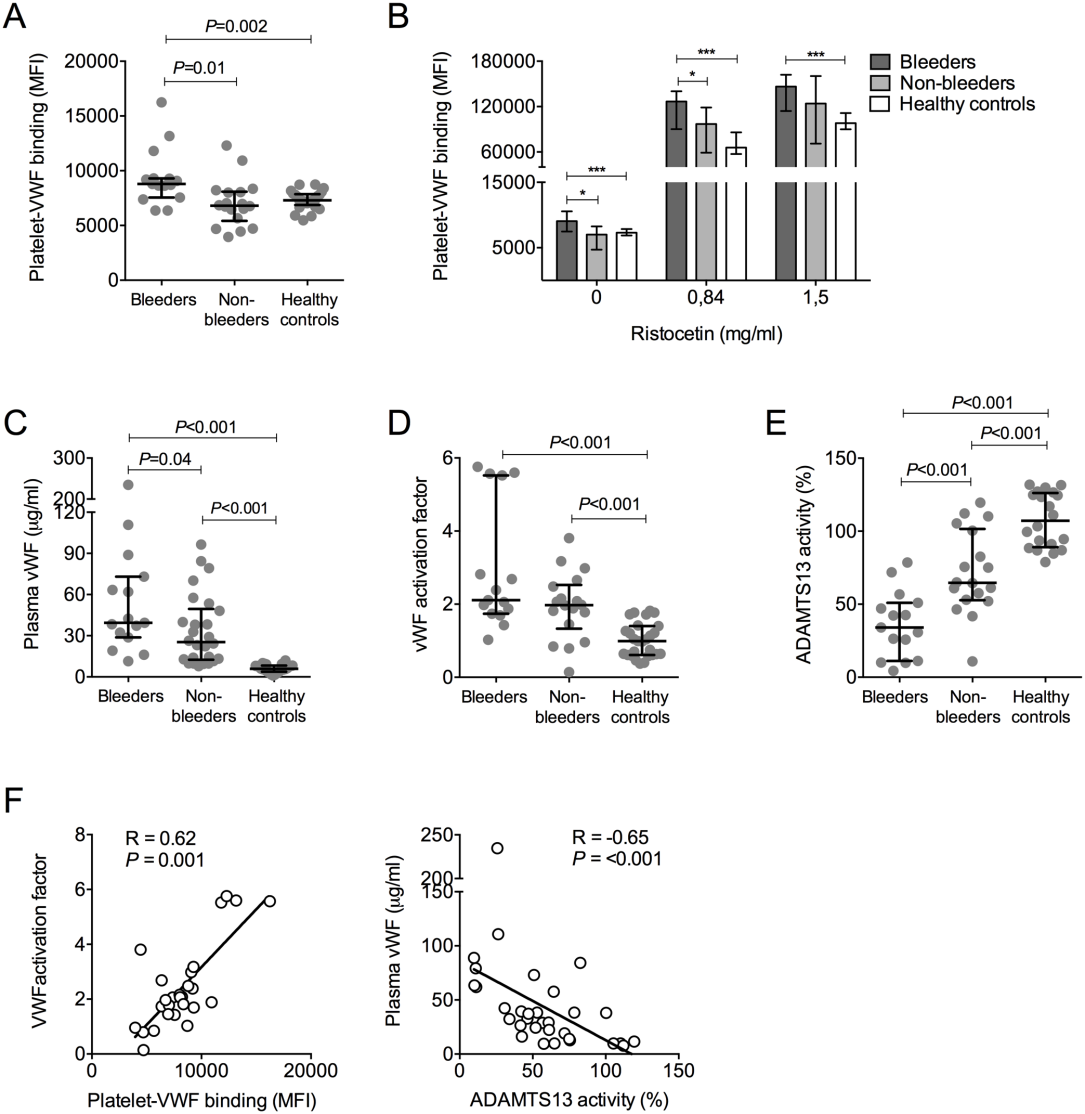
Platelet-von Willebrand factor (VWF) binding, plasma VWF levels, VWF activation factor and ADAMTS13 activity levels. Platelet-VWF binding (depicted as median fluorescence intensity (MFI) in arbitrary units) in unstimulated samples (A) and after *ex vivo* stimulation with two concentrations of ristocetin (B). * *P*<0.05, ***P<0.001. (C-E) Plasma concentrations of VWF, VWF activation factor and ADAMTS13 activity. Data are shown as medians with IQR. (F) Spearman correlation coefficient of VWF activation factor and platelet-VWF binding (left panel), as well as plasma VWF and ADAMTS13 activity (right panel) of leptospirosis patients with and without bleeding.

Binding of VWF to platelets is associated with platelet clearance, platelet activation and also possibly platelet dysfunction [15, 29]. We determined platelet-VWF binding using flow cytometry and measured plasma VWF levels, VWF activation factor and ADAMTS13 activity. Bleeders had a significantly higher platelet-VWF binding (MFI 9069, 7470–10554) than non-bleeders (MFI 6999, 4707–8250; *P* = 0.01) and healthy controls (MFI 7296, 6877–7867; *P* = 0.002) (Fig 3A). Upon activation of VWF by ristocetin, the bleeding patients demonstrated the highest increase in platelet-VWF binding (Fig 3B). These flow cytometric findings were consistent with the observation that bleeders had the highest plasma VWF concentration (Fig 3C) and that the VWF activation factor was about twofold higher in both the bleeding and non-bleeding leptospirosis patients than in healthy controls, indicating that a higher amount of the circulating VWF was in an active, platelet-binding conformation (Fig 3D).

ADAMTS13 functions as a natural regulator that de-activates the VWF by proteolysis [30] and can be consumed by high levels of circulating VWF [12]. ADAMTS13 activity levels were decreased in leptospirosis patients and were lowest in bleeders (Fig 3E). Three patients in the bleeders group had ADAMTS13 activity level lower than 10%. An ADAMTS13 activity below 50% was found in 11 (73%) patients in the bleeders group and only in three (25%) patients in the non-bleeders group. In the leptospirosis group as a whole, platelet-VWF binding correlated positively with the VWF activation factor (R = 0.62; *P* = 0.001; Fig 3F). Additionally, whereas ADAMTS13 activity did not correlate with VWF activation factor, it had an inverse correlation (R = -0.65; *P* = 0.0009) with plasma VWF levels, suggesting consumption of ADAMTS13 by VWF (data for correlation between ADAMTS13 and plasma VWF shown in Fig 3F). These correlations were consistent across the bleeders and non-bleeders group, as shown in S3A and S3B Fig.

Additional analyses of the MAT-positive and MAT-negative subgroups are presented in S4 Fig while data on follow-up measurements on day 4 are presented in S5 Fig.

### Platelet-vWF binding correlates with thrombocytopenia

**Fig 4:**
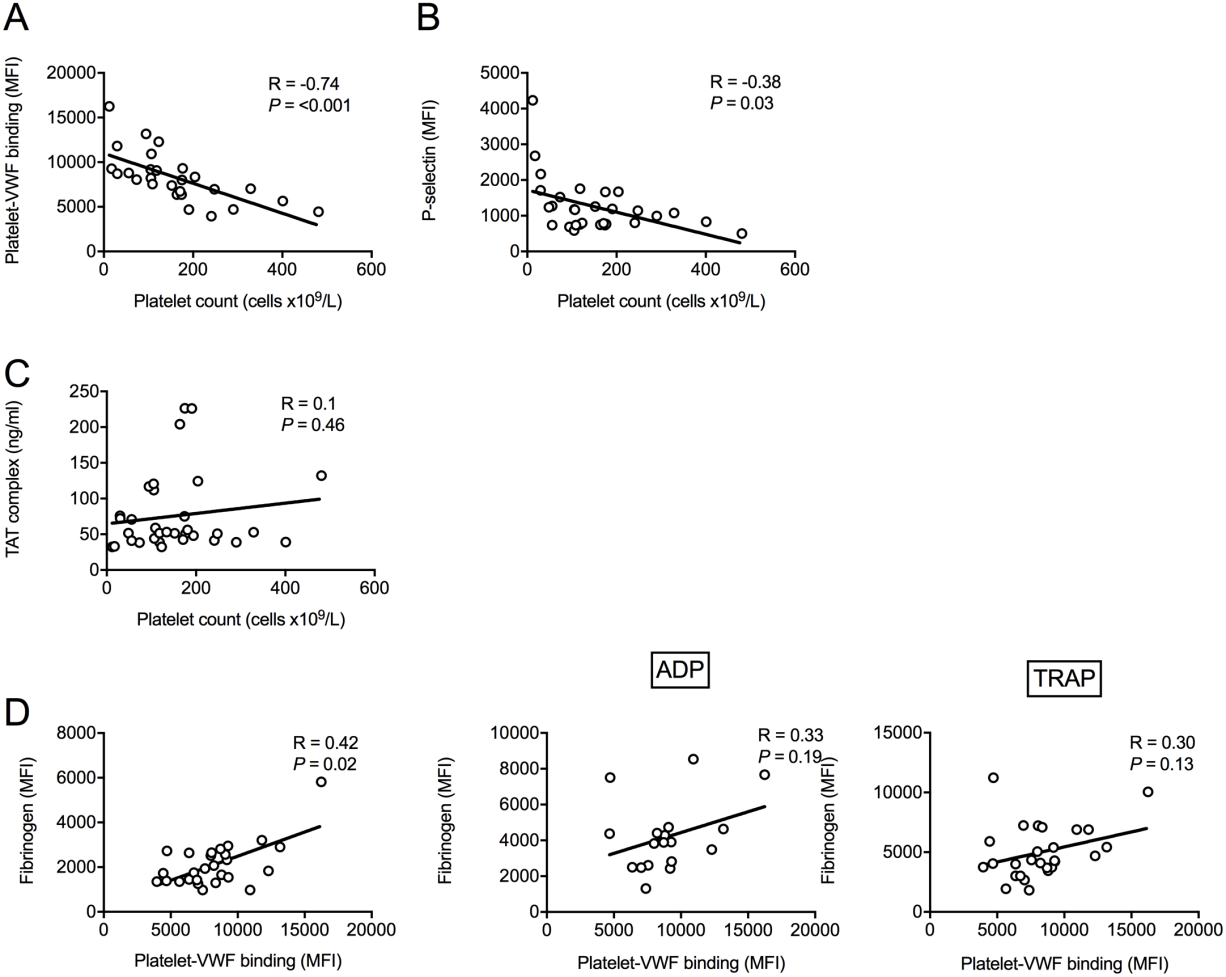
Platelet count and platelet-VWF binding correlates. (A-C) Spearman correlation of platelet count with platelet-VWF binding, platelet P-selectin expression and thrombin anti-thrombin complex. (D) Spearman correlation of platelet-VWF binding and platelet-fibrinogen binding in unstimulated sample (left panel) and upon ADP (125 μM) and TRAP (625 μM) stimulation (middle and right panel). Data for leptospirosis patients with and without bleeding are shown.

Next, we checked associations between parameters to explore possible mechanisms underlying the leptospirosis-associated thrombocytopenia and platelet dysfunction (Fig 4). First, there was a strong inverse correlation of platelet count with platelet-VWF binding and to a lesser extent with the platelet membrane expression of P-selectin (Fig 4A and 4B, day 4 data presented in S5 Fig). In contrast, plasma levels of the coagulation activation marker TAT complex did not correlate with platelet numbers (Fig 4C). Next, we explored whether platelet-VWF binding inhibited the platelet α_IIb_β_3_ signaling pathway, as recently reported for von Willebrand disease type 2B [15]. However, in contrast to our hypothesis, platelet-VWF binding correlated positively with platelet-fibrinogen binding in unstimulated samples (Fig 4D; Spearman R = 0.42; *P* = 0.02), and there was a trend for a positive correlation with high dose TRAP- and ADP-stimulated fibrinogen binding (R = 0.33; *P* = 0.19 for ADP and R = 0.30; *P* = 0.13 for TRAP).

Another possible cause of platelet dysfunction is uremia due to the presence of uremic toxins in the blood [31]. Although we did observe a trend for higher urea levels in bleeders compared to non-bleeders (*P* = 0.06), we did not find any associations between ureum levels with either P-selectin at baseline (Spearman R = -0.17, *P* = 0.36) or upon stimulation with ADP (R = -0.16, *P* = 0.43) or TRAP (R = -0.11, *P =* 0.58*)*

## Discussion

Our study reveals four important findings. First, platelet activation is a feature of severe, probable leptospirosis, especially in cases with bleeding manifestations. Second, bleeding complications are predominantly associated with platelet dysfunction rather than absolute platelet count. Third, circulating platelets in probable leptospirosis patients bind more VWF and this has a strong negative association with platelet number, in contrast with the plasma coagulation marker TAT complexes. Fourth, platelet-VWF binding did not diminish with agonist-induced platelet-fibrinogen binding (Fig 4D), disproving our hypothesis that increased platelet-VWF binding underlies the inhibition of the platelet α_IIb_β_3_ signaling pathway, as recently reported for von Willebrand disease type 2B [15].

To the best of our knowledge, this is the first study measuring platelet activation and reactivity in patients with probable leptospirosis. Platelet function studies are logistically challenging, as blood samples need to be processed without delay. In conditions with thrombocytopenia, aggregometry is also less reliable and flow cytometry-based assays are preferred [8]. The findings of our study are consistent with those from an experimental leptospirosis guinea pig model in which platelets were found in hepatic sinusoids [32] and in which thrombocytopenia was not related to disseminated intravascular coagulation. Another animal study, using a virulent serovar of *Leptospira interrogans* in gerbils, reported increased levels of platelet-activating factor acetylhydrolase (PAF-AH), which might contribute to inhibition of platelet activation [33]. Our observation that platelet function, rather than the absolute platelet count, determined the bleeding risk is consistent with increasing evidence from patients with immune thrombocytopenic purpura (ITP) that has identified platelet function as an important determinant of bleeding risk [34–36].

Some of the platelet parameters did not show statistically significant differences between the bleeding and non-bleeding groups. For example, platelet reactivity to ADP stimulation was lower in those with bleeding, but this did not reach statistical significance (Fig 1A). Except for the small sample size, lack of statistical difference may also reflect the fact that factors other than platelet dysfunction may contribute to bleeding, including inflammation, endotheliopathy and coagulopathy.

High plasma VWF levels, together with elevations in other endothelial cell activation markers, were recently reported in patients with leptospirosis [37]. Our findings add to this by showing that the circulating VWF is in an active, GPIbα-binding conformation, and that circulating platelets indeed have more VWF on their surface. Most VWF is derived from endothelial cells. However, platelets also contain VWF in their granules [38] and to what extent this contributes to the increased VWF binding on the platelet membrane is unknown. We also found a concurrent decrease in ADAMTS13 activity levels. This enzyme regulates the multimeric size and function of VWF through the cleavage of VWF within the A2 domain [39]. A severe reduction in ADAMTS13 activity as a result of auto-antibodies is a hallmark of the rare disease thrombotic thrombocytopenic purpura (TTP) [40]. Cases of leptospirosis-associated TTP have also been described, including that with severely reduced ADAMTS13 activity [41, 42]. Infections may lead to significant reductions in ADAMTS13 as a result of different mechanisms, as recently reviewed by Schwameis [43]. Multiple studies have shown that conditions with increased VWF release are associated with secondary ADAMTS13 consumption, such as in severe systemic infections [44] and after desmopressin-induced VWF release [45]. Inhibition of ADAMTS13 activity can also occur due to inflammation-induced IL-6 release [46] or proteolytic cleavage of ADAMTS13 by neutrophils [47]. And lastly, competition of ADAMTS13 with thrombospondin-1 for the interaction with the VWF-A3 domain may slow the proteolysis of ultra large (UL)-VWF multimers [43].

The mechanisms underlying the observed platelet activation and platelet dysfunction in our study population remain to be elucidated. It is unknown whether pathogenic *Leptospira interrogans* strains are able to directly interact with and activate platelets. Leptospiral lipopolysaccharide (LPS) was shown to be a ligand of Toll-like receptor (TLR)-2 and TLR4 in human whole blood and in mice [48, 49]. Platelets harbor both TLRs and the ligation of TLR-2 especially leads to a strong thrombotic platelet activating response [50]. Alternatively, increased platelet-VWF binding may activate platelets [51]. In patients with von Willebrand disease type 2B (VWD type 2B), a disease characterized by gain-of-function mutations in VWF that enhance its spontaneous binding to the platelet GPIbα, increased platelet-VWF binding is associated with thrombocytopathy due to inhibition of α_IIb_β_3_ activation [15]. We also hypothesized that this might underlie thrombocytopathy in leptospirosis, but this is unlikely with our finding of a positive correlation of platelet-VWF binding with fibrinogen binding to the activated α_IIb_β_3_ receptor in response to platelet agonists (Fig 4D). Another explanation might be that excessive platelet activation is responsible for less reactive and functional platelets. The exposure of platelets to inducers of platelet activation such as thrombin [52] or VWF [53] may lead to a partial release or incomplete degranulation of platelets, leading to impairment of their hemostatic effectiveness [16]. The combination of a higher platelet activation status of circulating platelets in the bleeding leptospirosis patients with reduced reactivity to *ex vivo* activation (Fig 1A) would certainly fit this hypothesis. A similar platelet phenotype was previously found in patients with other hemorrhagic infectious diseases, such as severe dengue [17]. In hantavirus-infected patients, platelet reactivity to agonist stimulation was significantly lower during disease compared to follow-up [54]. Impaired platelet dysfunction appears specific for these ‘hemorrhagic’ diseases and does not appear to result from more general factors such as inflammation, as most other infections increase platelet reactivity. For example, we found increased platelet reactivity using similar assays as employed in the current study in patients with sepsis due to common Gram-positive pathogens [55], pigs with pneumococcal bacteremia [56] and HIV-infected individuals on antiretroviral therapy [22]. In addition, we did not find systemic platelet activation in volunteers participating in a controlled human malaria infection, despite developing thrombocytopenia, which may explain why malaria is rarely complicated by bleeding [57].

There was a strong inverse correlation of VWF-platelet binding with platelet number, suggesting that VWF bound to the platelet membrane is involved in platelet clearance. Increased platelet-VWF interaction in VWD type 2B results in increased platelet clearance by the liver [58]. How the VWF-platelet complexes are being cleared remains to be determined. Evidence suggests that glycans on GPIbα are critical in mediating platelet clearance via receptors containing carbohydrate-binding domains on the macrophage αMβ2 integrin and the hepatic Ashwell-Morell receptor [29, 59, 60]. One plausible mechanistic explanation is that the increased platelet-VWF binding results in a structural unfolding of the GPIbα extracellular domain and triggers signaling into the platelet, desialylation of the platelet surface and platelet clearance [61].

The limitations of our study include the small number of patients tested positive with the MAT and the fact that patients were included solely based on a positive result of a rapid IgM lateral flow test combined with clinical manifestations consistent with leptospirosis, classifying the cases as probable leptospirosis. Individuals in the control group were all IgM lateral flow negative. The diagnosis of leptospirosis is challenging with current gold standard tests such as the MAT being imperfect and technically demanding [19]. At the time of the study, other diagnostic tests such as an IgM ELISA or PCR were not available in our setting. It is also uncertain whether PCR would have yielded a high sensitivity in our cohort as most cases were enrolled only in the second week of illness when the PCR is frequently already negative. The low number of patients with a positive MAT might be explained by the fact that the MAT also has limited sensitivity [19]. MAT gives a large number of false negative results in the early course of infection, as IgM antibodies detectable by MAT only appear after day 8 of illness and reach the peak by week 4 [62, 63]. MAT requires collection of paired sera at appropriate time intervals for the most accurate interpretation of results. Therefore, although it is of high value for epidemiological purposes, its value in the acute clinical setting is limited [64]. Many cases in our study could thus only be classified as probable leptospirosis. Subgroup analysis, however, did not show any differences in platelet parameters between those with a positive or negative result of the MAT. In addition, even though leptospirosis is a common cause of undifferentiated fever in Semarang, Indonesia, where our study was performed, we cannot exclude other co-infections, such as murine typhus [65].

In conclusion, we found that platelet activation and platelet dysfunction are features of patients with probable leptospirosis diagnosis that are associated with the severity of bleeding events. Circulating platelets also bind more with VWF, and although this does not explain the observed platelet dysfunction, it may play a role in platelet activation and clearance. Bleeding is a serious, life-threatening complication of leptospirosis and our findings warrant further study on the clinical utility of platelet function tests, as it is thrombocytopathy rather than thrombocytopenia that is associated with the severity of bleeding events. In severe bleeding, platelet transfusion may temporarily reverse platelet dysfunction. In addition, given the presumed role of excessive VWF-platelet interaction in thrombocytopenia in severe leptospirosis, novel therapies aimed at preventing this interaction, including recombinant ADAMTS13 [66], might also have some therapeutic value. This needs to be addressed in future studies.

## Supporting information

S1 Fig Correlations between bleeding scale and ADP-induced (125 μM) P-selectin expression (A) and platelet-fibrinogen binding (B). Shown are the Spearman correlation coefficients.
(PDF)

S2 Fig Platelet activation and reactivity upon follow-up at day four. (A) Platelet-fibrinogen binding and the platelet membrane expression of P-selectin (depicted as median fluorescence intensity (MFI) in arbitrary units) in unstimulated samples and after stimulation with two concentrations of the platelet agonists, thrombin receptor activating peptide (TRAP) and adenosine diphosphate (ADP), in leptospirosis patients with bleeding (bleeders, n = 15) and without bleeding (non-bleeders, n = 18) and in healthy controls (n = 25). * *P*<0.05, ***P<0.005. (B) Plasma concentration of soluble P-selectin. Data depicted are medians with IQR. Data of patients were from day 4, while data from healthy controls were from day 1.
(PDF)

S3 Fig Relationship between platelet-von Willebrand factor (VWF) binding and VWF activation factor as well as ADAMTS13 and plasma VWF levels. (A) Spearman correlation coefficient of VWF activation factor and platelet-VWF binding in the bleeders and non-bleeders. (B) Spearman correlation coefficient of plasma VWF and ADAMTS13 activity of bleeders and non-bleeders.
(PDF)

S4 Fig Platelet-von Willebrand factor (VWF) binding in patient groups based on microscopic agglutination test (MAT) results. Platelet-VWF binding (depicted as median fluorescence intensity (MFI) in arbitrary units) in unstimulated samples (A) and after *ex vivo* stimulation with two concentrations of ristocetin (B). **P<0.005.
(PDF)

S5 Fig Platelet-von Willebrand factor (VWF) binding, plasma VWF, VWF activation factor and platelet count correlates upon follow-up at day 4. (A) Platelet-VWF binding (depicted as median fluorescence intensity (MFI) in arbitrary units) in unstimulated samples and after *ex vivo* stimulation with two concentrations of ristocetin. (B-C) Plasma concentrations of VWF and VWF activation factor. (D) Spearman correlation coefficient of platelet count and platelet-VWF binding as well as platelet P-selectin expression. Data are shown as medians with IQR. Data from patients were from day 4, while data from healthy controls were from day 1.
(PDF)
