# Supplementary material for: Platelet dysfunction contributes to bleeding complications in patients with probable leptospirosis
Source: PLoS Negl Trop Dis. 2017 Sep 21;11(9):e0005915. doi: 10.1371/journal.pntd.0005915 (PMC5626517; doi:10.1371/journal.pntd.0005915)
Supplement: S1 Fig — Shown are the Spearman correlation coefficients. (PDF) [file pntd.0005915.s001.pdf]

S1 Fig

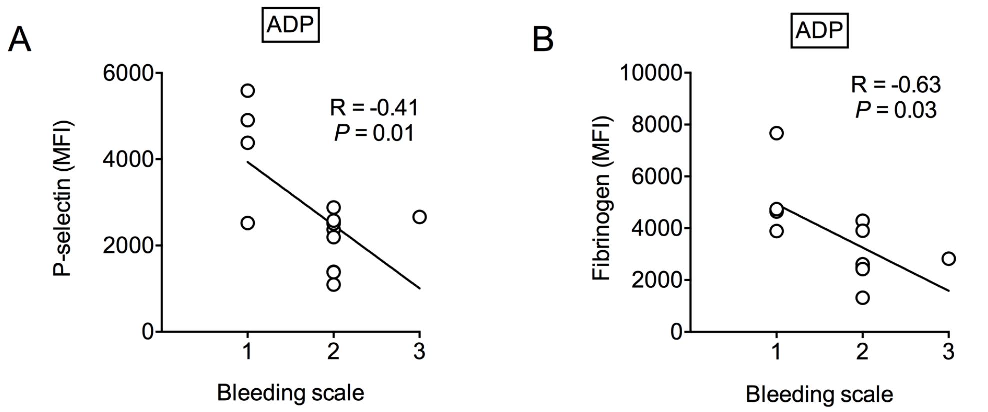

S1 Fig. Correlations between bleeding scale and ADP-induced (125 μM) P-selectin expression (A) and platelet-fibrinogen binding (B). Shown are the Spearman correlation coefficients.
