# Supplementary material for: Platelet dysfunction contributes to bleeding complications in patients with probable leptospirosis
Source: PLoS Negl Trop Dis. 2017 Sep 21;11(9):e0005915. doi: 10.1371/journal.pntd.0005915 (PMC5626517; doi:10.1371/journal.pntd.0005915)
Supplement: S2 Fig — (A) Platelet-fibrinogen binding and the platelet membrane expression of P-selectin (depicted as median fluorescence intensity (MFI) in arbitrary units) in unstimulated samples and after stimulation with two concentrations of the platelet agonists, thrombin receptor activating peptide (TRAP) and adenosine diphosphate (ADP), in leptospirosis patients with bleeding (bleeders, n = 15) and without bleeding (non-bleeders, n = 18) and in healthy controls (n = 25). * P<0.05, ***P<0.005. (B) Plasma concentration of soluble P-selectin. Data depicted are medians with IQR. Data of patients were from day 4, while data from healthy controls were from day 1. (PDF) [file pntd.0005915.s002.pdf]

S2 Fig

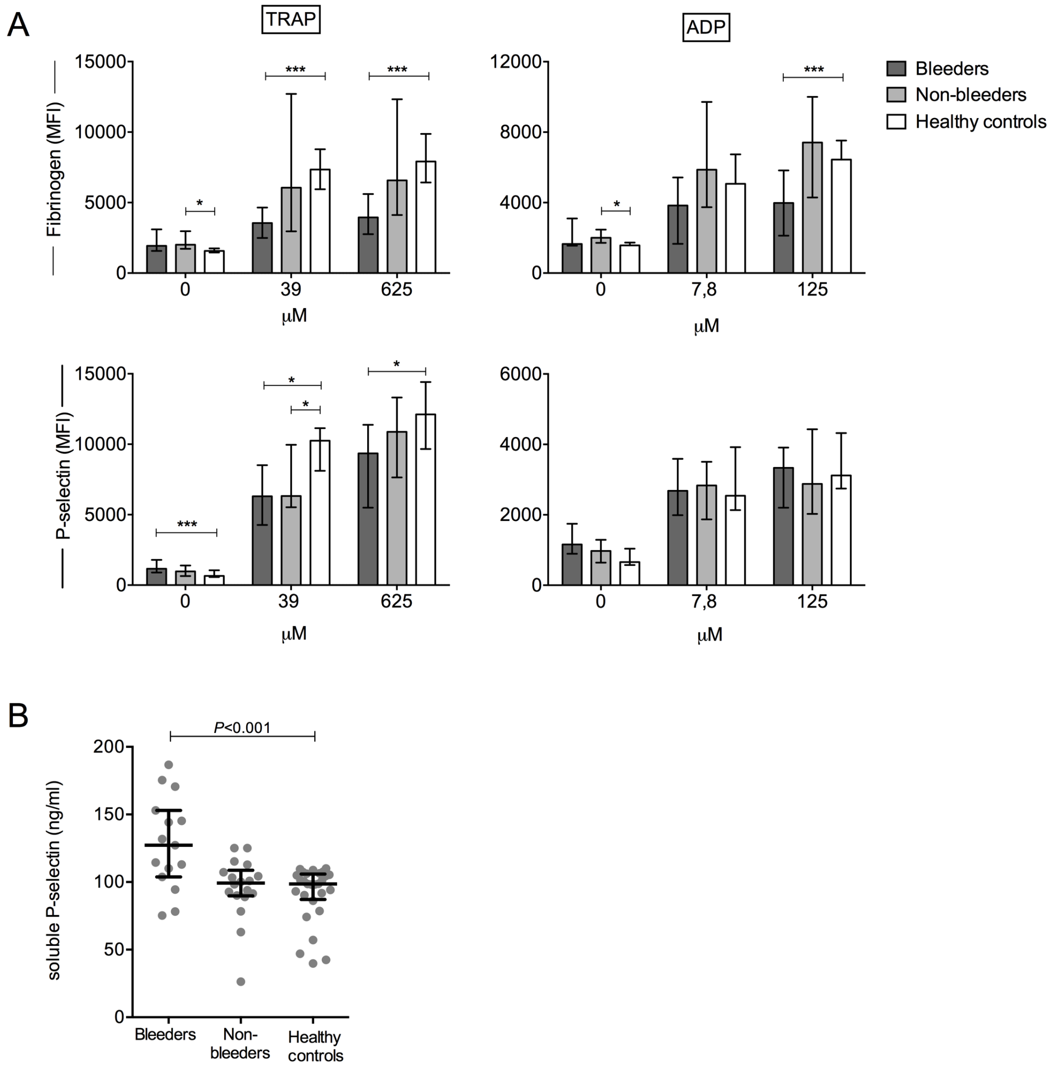

S2 Fig. Platelet activation and reactivity upon follow-up at day four. (A) Platelet-fibrinogen binding and the platelet membrane expression of P-selectin (depicted as median fluorescence intensity (MFI) in arbitrary units) in unstimulated samples and after stimulation with two concentrations of the platelet agonists, thrombin receptor activating peptide (TRAP) and adenosine diphosphate (ADP), in leptospirosis patients with bleeding (bleeders, n=15) and without bleeding (non-bleeders, n=18) and in healthy controls (n=25). * *P*<0.05, ****P*<0.005. (B) Plasma concentration of soluble P-selectin. Data depicted are medians with IQR. Data of patients were from day 4, while data from healthy controls were from day 1.
