# Supplementary material for: Platelet dysfunction contributes to bleeding complications in patients with probable leptospirosis
Source: PLoS Negl Trop Dis. 2017 Sep 21;11(9):e0005915. doi: 10.1371/journal.pntd.0005915 (PMC5626517; doi:10.1371/journal.pntd.0005915)
Supplement: S3 Fig — (A) Spearman correlation coefficient of VWF activation factor and platelet-VWF binding in the bleeders and non-bleeders. (B) Spearman correlation coefficient of plasma VWF and ADAMTS13 activity of bleeders and non-bleeders. (PDF) [file pntd.0005915.s003.pdf]

S3 Fig

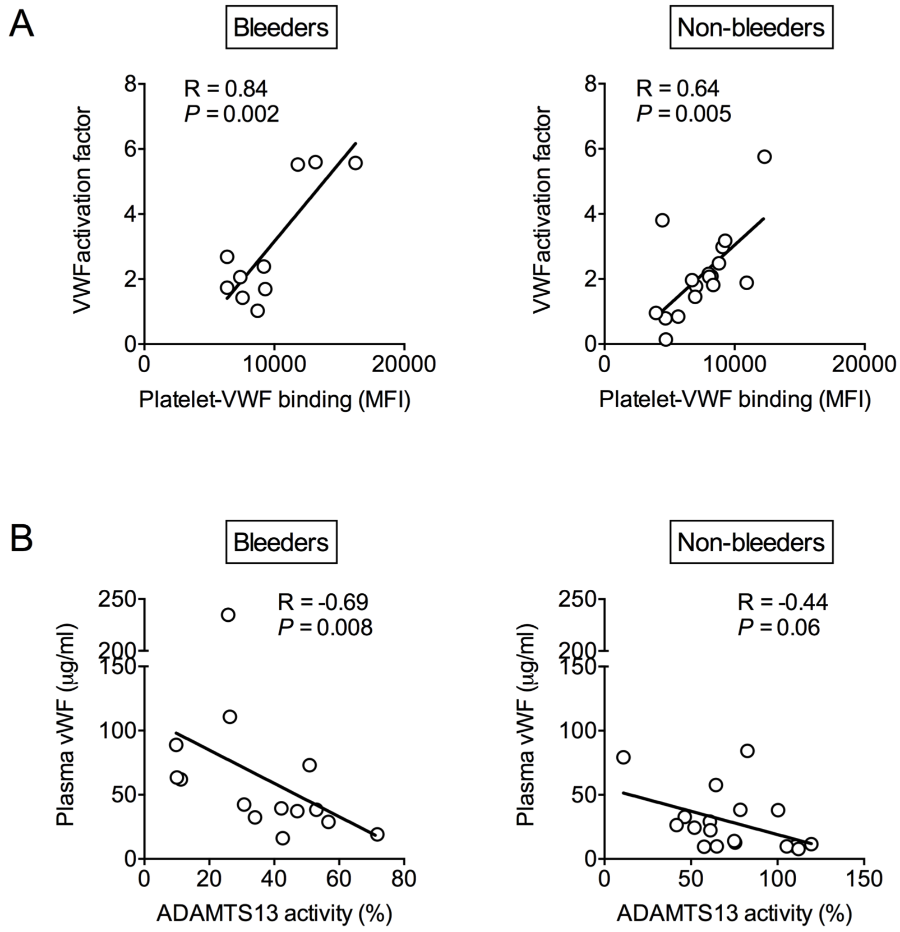

S3 Fig. Relationship between platelet-von Willebrand factor (VWF) binding and VWF activation factor as well as ADAMTS13 and plasma VWF levels. (A) Spearman correlation coefficient of VWF activation factor and platelet-VWF binding in the bleeders and non-bleeders. (B) Spearman correlation coefficient of plasma VWF and ADAMTS13 activity of bleeders and non-bleeders.
