# Supplementary material for: Platelet dysfunction contributes to bleeding complications in patients with probable leptospirosis
Source: PLoS Negl Trop Dis. 2017 Sep 21;11(9):e0005915. doi: 10.1371/journal.pntd.0005915 (PMC5626517; doi:10.1371/journal.pntd.0005915)
Supplement: S4 Fig — Platelet-VWF binding (depicted as median fluorescence intensity (MFI) in arbitrary units) in unstimulated samples (A) and after ex vivo stimulation with two concentrations of ristocetin (B). **P<0.005. (PDF) [file pntd.0005915.s004.pdf]

S4 Fig

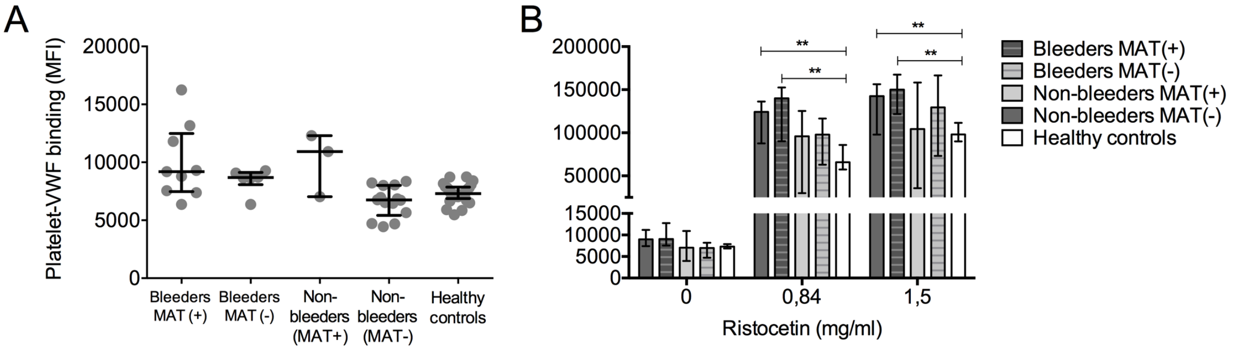

S4 Fig. Platelet-von Willebrand factor (VWF) binding in patient groups based on microscopic agglutination test (MAT) results. Platelet-VWF binding (depicted as median fluorescence intensity (MFI) in arbitrary units) in unstimulated samples (A) and after *ex vivo* stimulation with two concentrations of ristocetin (B). ***P*<0.005.
