# Supplementary material for: Platelet dysfunction contributes to bleeding complications in patients with probable leptospirosis
Source: PLoS Negl Trop Dis. 2017 Sep 21;11(9):e0005915. doi: 10.1371/journal.pntd.0005915 (PMC5626517; doi:10.1371/journal.pntd.0005915)
Supplement: S5 Fig — (A) Platelet-VWF binding (depicted as median fluorescence intensity (MFI) in arbitrary units) in unstimulated samples and after ex vivo stimulation with two concentrations of ristocetin. (B-C) Plasma concentrations of VWF and VWF activation factor. (D) Spearman correlation coefficient of platelet count and platelet-VWF binding as well as platelet P-selectin expression. Data are shown as medians with IQR. Data from patients were from day 4, while data from healthy controls were from day 1. (PDF) [file pntd.0005915.s005.pdf]

S5 Fig

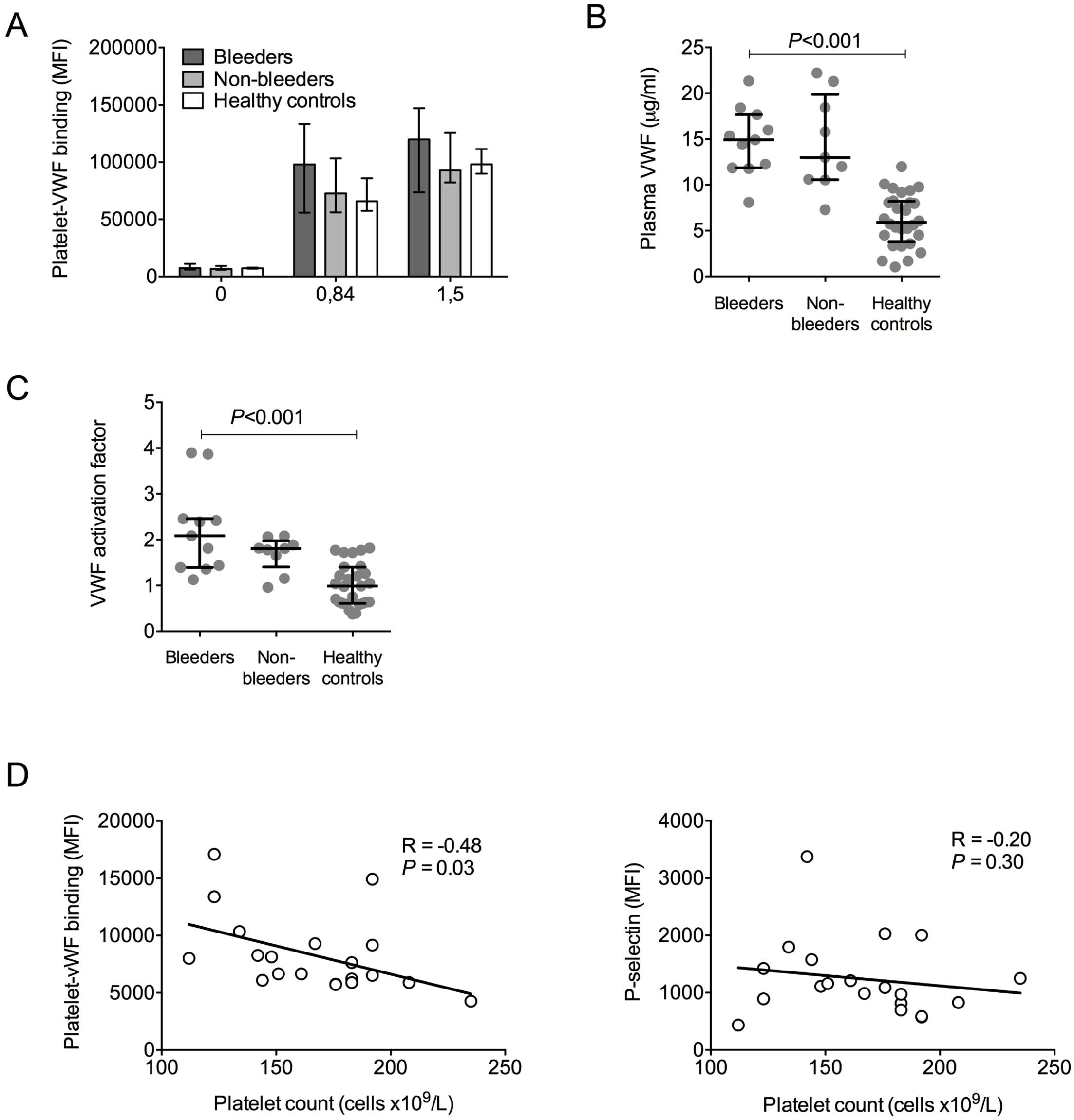

S5 Fig. Platelet-von Willebrand factor (VWF) binding, plasma VWF, VWF activation factor and platelet count correlates upon follow-up at day 4. (A) Platelet-VWF binding (depicted as median fluorescence intensity (MFI) in arbitrary units) in unstimulated samples and after *ex vivo* stimulation with two concentrations of ristocetin. (B-C) Plasma concentrations of VWF and VWF activation factor. (D) Spearman correlation coefficient of platelet count and platelet-VWF binding as well as platelet P-selectin expression. Data are shown as medians with IQR. Data from patients were from day 4, while data from healthy controls were from day 1.
